# Taylor–Couette and related flows on the centennial of Taylor’s seminal *Philosophical Transactions* paper: part 2

**DOI:** 10.1098/rsta.2022.0359

**Published:** 2023-05-01

**Authors:** Rainer Hollerbach, Richard M. Lueptow, Eric Serre

**Affiliations:** ^1^ School of Mathematics, University of Leeds, Leeds LS2 9JT, UK; ^2^ Department of Mechanical Engineering, Northwestern University, Evanston, IL 60208, USA; ^3^ M2P2, Aix Marseille University, Cent. Marseille, CNRS, Marseille, France

**Keywords:** Taylor–Couette flow, stability analysis

## Abstract

In 1923, the *Philosophical Transactions* published G. I. Taylor’s seminal paper on the stability of what we now call Taylor–Couette flow. In the century since the paper was published, Taylor’s ground-breaking linear stability analysis of fluid flow between two rotating cylinders has had an enormous impact on the field of fluid mechanics. The paper’s influence has extended to general rotating flows, geophysical flows and astrophysical flows, not to mention its significance in firmly establishing several foundational concepts in fluid mechanics that are now broadly accepted. This two-part issue includes review articles and research articles spanning a broad range of contemporary research areas, all rooted in Taylor’s landmark paper.

This article is part of the theme issue ‘Taylor–Couette and related flows on the centennial of Taylor’s seminal *Philosophical Transactions* paper (part 2)’.

In a remarkable paper published in the *Philosophical Transactions A* a century ago, G. I. Taylor connected theory and experiment in his ground-breaking investigation of flow between differentially rotating concentric cylinders [1]. The paper is sometimes described as the first convincing proof of the applicability of mathematical approaches to predict stability as well as proof of the fundamental correctness of the Navier–Stokes equations and the no-slip boundary condition [2], which are foundational concepts in modern fluid mechanics.

Taylor’s 1923 paper has inspired several generations of researchers in fields ranging from nonlinear dynamics to astrophysics. Not only does Taylor–Couette flow display remarkable vortical patterns that are easily generated and visualized, it is a test bed for studies probing fundamental aspects of fluid flow as well as practical engineering applications.

Part 1 of this two-part theme issue explored contemporary topics related to Taylor–Couette flow including turbulent, convective and two-phase flows as well as extensions to magnetohydrodynamic, ferrofluidic and viscoelastic flows and flow geometries that are closely related to the Taylor–Couette problem [3–18]. Part 2 of this theme issue continues with review articles and research articles having their origin in Taylor’s 1923 paper. Like Part 1, contributions in Part 2 come from an international community of leading researchers studying fluid flows that all connect back to Taylor’s 1923 pioneering work published in the *Philosophical Transactions*.

This issue starts with several papers that consider much the same problem as Taylor did, but with an emphasis on turbulence rather than the linear onset of instability. Feldmann *et al.* [19] review the different routes to turbulence in the classical problem. Wang *et al.* [20] present numerical results in the supercritical turbulent spiral regime. Merbold *et al.* [21] conduct experiments in a relatively unexplored parameter space, namely the very wide gap case with cylindrical radius ratio $r_{i}/r_{o}=0.1$. Most of their resulting flow states are also turbulent.

As in Part 1, we have several papers on the interaction of Taylor–Couette flow with convection, but with one crucial difference. The papers in Part 1 imposed a temperature gradient in the cylindrically radial direction, which corresponds to a radial force field playing a role like that of gravity. By contrast, the papers here impose gradients in the axial direction, matching gravity in a typical laboratory setting. Lopez *et al.* [22] review Taylor–Couette flows in stably stratified configurations. Closely related to this is the paper by Meletti *et al.* [23], who present numerical work on the so-called strato-rotational instability. This is an intriguing phenomenon whereby the imposed differential rotation can be in the Rayleigh-stable regime, and the stratification by itself would also be stable, but the combination of the two is nevertheless unstable. By contrast, Masuda *et al.* [24] impose unstable axial temperature gradients, and then study how the resulting convection interacts with the underlying Taylor–Couette flows.

Ji & Goodman [25] present a review of magnetohydrodynamic Taylor–Couette flows, with a particular emphasis on the magnetorotational instability and attempts to obtain various versions of it in liquid metal experiments. Both the magnetorotational instability and the strato-rotational instability are believed to be important in understanding the dynamics of astrophysical accretion discs, so experiments specifically targeting this regime are of considerable interest.

As was the case in Part 1, the Taylor–Couette problem can be extended by considering fluids that are more complex than Newtonian fluids. Bai *et al.* [26] and Moazzen *et al.* [27] both present experimental results using viscoelastic fluids. Lopez & Altmeyer [28] consider viscoelastic fluids numerically, and obtain arrow-shaped rotating waves. Panwar *et al.* [29] conduct experiments involving oil-in-water emulsions in Taylor–Couette flows. Kang & Mirbod [30] present a numerical study of non-colloidal suspensions in a Taylor–Couette flow.

Finally, and again as in Part 1, there are a variety of systems and geometries that are not strictly Taylor–Couette flows as such, but are nevertheless closely related. Nagata [31] presents theoretical comparisons of very narrow gap Taylor–Couette flow and rotating plane Couette flow. Krivonosova *et al.* [32] consider spherical Couette flow, the flow between differentially rotating spheres. After a brief review of the subject, they investigate the effect of noise on some of the resulting flow states. Sharma *et al.* [33] numerically study two lid-driven flow systems and confirm the presence of a Taylor–Görtler-like instability in those geometries.

This two-part theme issue is a fitting tribute to celebrate the centennial of Taylor’s foundational paper in the *Philosophical Transactions*. It is clear that the study of Taylor–Couette flow will continue to provide a basis for a broad range of important and fundamental research topics for many decades to come.

## Data Availability

This article has no additional data.
